# Allele-specific locus binding and genome editing by CRISPR at the *p16INK4a* locus

**DOI:** 10.1038/srep30485

**Published:** 2016-07-28

**Authors:** Toshitsugu Fujita, Miyuki Yuno, Hodaka Fujii

**Affiliations:** 1 Chromatin Biochemistry Research Group, Combined Program on Microbiology and Immunology, Research Institute for Microbial Diseases, Osaka University, 3-1 Yamadaoka, Suita, 565-0871 Osaka, Japan

## Abstract

The clustered regularly interspaced short palindromic repeats (CRISPR) system has been adopted for a wide range of biological applications including genome editing. In some cases, dissection of genome functions requires allele-specific genome editing, but the use of CRISPR for this purpose has not been studied in detail. In this study, using the *p16INK4a* gene in HCT116 as a model locus, we investigated whether chromatin states, such as CpG methylation, or a single-nucleotide gap form in a target site can be exploited for allele-specific locus binding and genome editing by CRISPR *in vivo*. First, we showed that allele-specific locus binding and genome editing could be achieved by targeting allele-specific CpG-methylated regions, which was successful for one, but not all guide RNAs. In this regard, molecular basis underlying the success remains elusive at this stage. Next, we demonstrated that an allele-specific single-nucleotide gap form could be employed for allele-specific locus binding and genome editing by CRISPR, although it was important to avoid CRISPR tolerance of a single nucleotide mismatch brought about by mismatched base skipping. Our results provide information that might be useful for applications of CRISPR in studies of allele-specific functions in the genomes.

Genome editing is performed widely in biological research. Engineered DNA-binding molecules such as zinc finger proteins, transcription activator-like effector (TAL or TALE) proteins, and the clustered regularly interspaced short palindromic repeats (CRISPR) system have been used for efficient genome editing^1^,^2^,^3^,^4^,^5^,^6^,^7^,^8^. Among these engineered DNA-binding molecules, CRISPR is the most convenient, economical, and time-efficient tool; consequently, it has been widely adopted in genome editing. This system can also be used for a wide range of biological applications such as artificial transcriptional regulation^2^,^5^,^6^,^9^, epigenetic modification^9^, locus imaging^5^,^6^,^9^, and isolation of specific genomic regions in a locus-specific manner^5^,^9^,^10^. In these applications, a catalytically inactive form of Cas9 (dCas9) fused to factors such as transcriptional regulators or epigenetic modifiers can be employed for locus-specific binding.

In most cases of genome editing, as well as in many other applications of CRISPR, both the maternal and paternal alleles of a given locus are targeted. By contrast, allele-specific targeting is occasionally required in studies of phenomena such as X-chromosome inactivation, genomic imprinting, and cancer, in which some loci are epigenetically regulated in an allele-specific manner^11^,^12^,^13^. In this regard, it is possible to exploit allelic differences in DNA sequences to achieve allele-specific genome editing. Indeed, allelic single-nucleotide polymorphisms (SNPs) in target sequences have been used in allele-specific CRISPR-mediated genome editing^14^,^15^. By contrast, it remains unclear whether an allele-specific single-nucleotide insertion/deletion (indel) mutation, mentioned hereafter as “single-nucleotide gap form”, can also be utilized for this purpose and other CRISPR applications.

It may also be possible to take advantage of allele-specific differences in chromatin states, such as DNA and histone modifications, in applications of CRISPR. For example, in genomic imprinting, one allele of a locus is in an open chromatin state and transcribed, whereas the other allele is closed by heterochromatinization induced by DNA or histone modifications^12^,^16^,^17^,^18^. In such cases, genome editing could be preferentially introduced into the accessible open allele. Alternatively, DNA or histone modifications at target sites might directly affect genome editing by CRISPR. Although CRISPR can edit CpG-methylated sequences *in vivo* and *in vitro*^19^, it remains unclear whether CpG methylation can be used for allele-specific locus binding and genome editing. If the CRISPR system shows binding preference to a CpG-methylated target site or an unmethylated one, this property could be exploited in allele-specific CRISPR applications.

In this study, using the *p16INK4a* gene in HCT116 as a model locus, we investigated whether different chromatin states or a single-nucleotide gap form at target sites can be exploited for allele-specific CRISPR applications *in vivo*. We showed that allele-specific targeting of CpG-methylated regions could be achieved with one of six guide RNAs (gRNAs) tested. The allelic specificity was not determined by CpG methylation. In addition, we showed that a single-nucleotide gap form in one allele could be exploited for allele-specific locus binding and genome editing by CRISPR. Our results might facilitate applications of CRISPR to studies of allele-specific genome functions.

## Results

### Allele-specific genome editing using the CRISPR complex at CpG-methylated target regions *in vivo*

**Figure 1:**
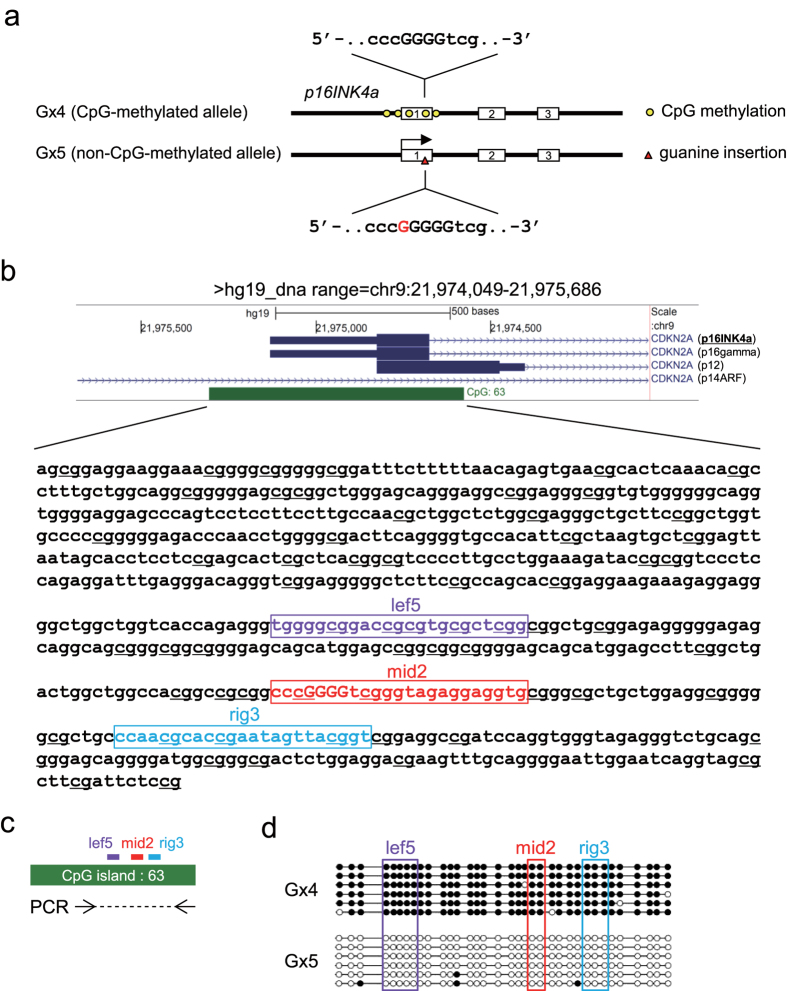
Structure of the human *p16INK4a* gene in HCT116. (**a**) The Gx4 allele is not transcribed because the CpG island (including the promoter region, first exon, and first intron) is CpG-methylated. In the Gx5 allele, a frameshift mutation caused by insertion of a single guanine (G, shown in red) in the coding region of the first exon prevents production of the functional protein. The Gx4 and Gx5 sequences are shown in uppercase. (**b**) The CpG island of the *p16INK4a* gene. (Upper) Schematic diagram of the CpG island around the first exon of *p16INK4a*. Four alternatively spliced mRNAs are transcribed from the *CDKN2A* locus, one of which is *p16INK4a*. The CpG island is shown in green. (Lower) DNA sequence of the CpG island in the Gx4 allele. An additional guanine (G) is inserted into the G stretch (shown in uppercase) of the Gx5 allele. The upper image and DNA sequence were generated using the UCSC Genome Browser (https://genome.ucsc.edu/). CpG sites are underlined. (**c**) Primer positions for bisulfite sequencing. (**d**) Bisulfite sequencing of genomic DNA extracted from HCT116. The target sites for sgRNA_lef5, sgRNA_mid2, and sgRNA_rig3 are shown in purple, red, and light blue, respectively (**b**–**d**).

In the human colorectal carcinoma cell line HCT116, one allele of the *p16INK4a* gene is not transcribed due to heavy methylation of the associated CpG island, which extends from the promoter to the first intron (Fig. 1)^20^,^21^,^22^,^23^, and contains H3K9m2, a heterochromatin mark^24^. The other allele, which is not CpG-methylated, is transcribed; however, it bears a single-guanine insertion in the first exon, resulting in a frameshift mutation that prevents production of a functional protein (Fig. 1a)^20^,^21^,^22^,^23^. These properties of the *p16INK4a* locus in HCT116 make this cell line ideal for investigating whether chromatin states and a single-nucleotide gap form can be utilized for allele-specific CRISPR-mediated locus binding and genome editing *in vivo*.

**Figure 2:**
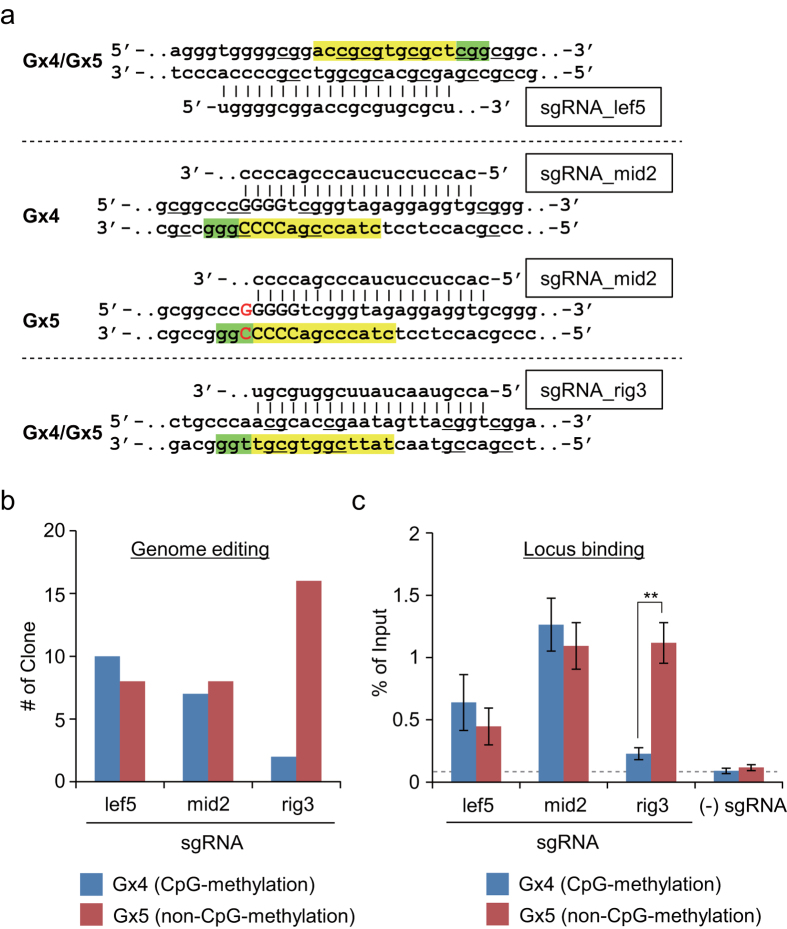
Effects of CpG methylation of target sites on genome editing *in vivo*. (**a**) DNA sequences targeted by sgRNAs. Seed sequences and PAMs are shown in yellow and green, respectively. The single-guanine insertion in the Gx5 allele is shown in red. CpG sites in the Gx4 allele are underlined. (**b**) Evaluation of genome editing. Schemes for genome editing and genotyping PCR are shown in Supplementary Fig. S1. Products of genotyping PCR were cloned, and 15 (sgRNA_mid2) or 18 (sgRNA_lef5 and sgRNA_rig3) independent clones were subjected to DNA sequencing analysis to identify the targeted alleles. (**c**) Evaluation of locus binding, as determined by DNA yields of enChIP. Error bars represent s.e.m. of three enChIP experiments (**t-test P-value < 0.01).

First, to examine feasibility of allele-specific genome editing, we designed three chimeric single gRNAs (sgRNAs) that included different numbers of CpGs, namely sgRNA_lef5, sgRNA_mid2, and sgRNA_rig3, which targeted genomic sequences containing five, two, and three CpGs, respectively, in the CpG island of *p16INK4a* (Figs 1b–d and 2a). In addition, they contained four, two, and two CpGs, respectively, in the seed sequence and/or protospacer adjacent motif (PAM) (Fig. 2a), positions that determine recognition of target sequences by CRISPR^25^.

Using these sgRNAs, we investigated whether allele-specific genome editing can be achieved by targeting allele-specifically transcribed locus *in vivo*. We transfected wild-type Cas9 and sgRNA expression plasmids, along with donor single-strand DNA (ssDNA), into wild-type HCT116 or HCT116-derived HCT116/del#3 cells to induce knock-in by homologous recombination (Supplementary Figs S1 and S2). We analyzed the outcome by genotyping PCR followed by DNA cloning and sequencing (Supplementary Fig. S1); the allele-specific single-guanine insertion (wild-type HCT116) or 31 nt deletion (HCT116/del#3) in the first exon of the *p16INK4a* gene can be used to distinguish the corresponding alleles by DNA sequencing. As shown in Fig. 2b, when sgRNA_lef5 or sgRNA_mid2 was used, the efficiencies of genome editing were comparable for both alleles. By contrast, when sgRNA_rig3 was used, the intended mutation was introduced preferentially in the non-CpG-methylated Gx5 allele (Fig. 2b). These results suggest that CRISPR-mediated genome editing is not necessarily affected by CpG methylation *in vivo*. However, the results obtained with sgRNA_rig3 show a possibility that allele-specific genome editing can be achieved by targeting an imprinted locus.

Table 1 summarizes information on CpG positions at target sites. Our results suggest that the allelic preference of sgRNA_rig3 was not related to the level of CpG methylation per se, because sgRNA_lef5 (which targets more CpGs) had no allelic preference. Moreover, sgRNA_lef5 and sgRNA_mid2 contain four and two CpGs, respectively, in their seed sequences and PAMs, but did not exhibit allelic preferences, whereas sgRNA_rig3, which has two CpGs in these sequences, did exhibit a preference. Therefore, the allelic preference of a given sgRNA might not be related to the number of CpGs in the seed sequence and/or PAM, but might instead simply be determined by the local accessibility of target sites.

### Allele-specific locus binding of the CRISPR complex in CpG-methylated target regions *in vivo*

To explore this idea, we examined the locus accessibility of CpG-methylated target sites using a CRISPR complex consisting of dCas9 and sgRNA *in vivo*. We developed engineered DNA-binding molecule-mediated chromatin immunoprecipitation (enChIP) technology using dCas9 for identification of molecules that interact with genomic regions of interest *in vivo*^26^,^27^,^28^,^29^,^30^,^31^ (see review^10^). In enChIP, sgRNA and dCas9 (fused to an epitope tag, if necessary) expressed in cells bind to a locus specified by the sequence of the sgRNA. The targeted locus can be isolated by affinity purification using an antibody (Ab) against the epitope tag or dCas9 itself (Supplementary Fig. S3a). In this study, we quantitatively evaluated allele-specific binding of the CRISPR complex to the target sites by enChIP followed by bisulfite treatment and quantitative methylation-specific PCR (MSP) (Supplementary Fig. S3a). The primer set designed for MSP could clearly distinguish CpG-methylated and non-CpG-methylated alleles (Supplementary Fig. S3b,c). As shown in Fig. 2c, enChIP with sgRNA_lef5 or sgRNA_mid2 resulted in a comparable percentage of input (DNA yield) between the CpG-methylated Gx4 and non-CpG-methylated Gx5 alleles, suggesting that CpG methylation had no effect on binding of the CRISPR complex to these loci *in vivo*. By contrast, enChIP with sgRNA_rig3 resulted in significantly higher DNA yields for the non-CpG-methylated Gx5 allele. These findings are consistent with the results of genome editing (Fig. 2b and Table 1), suggesting that the allelic preference of genome editing reflects the accessibility of a locus to the CRISPR complex.

### CpG methylation does not directly suppress binding of the CRISPR complex to purified DNAs *in vitro*

**Figure 3:**
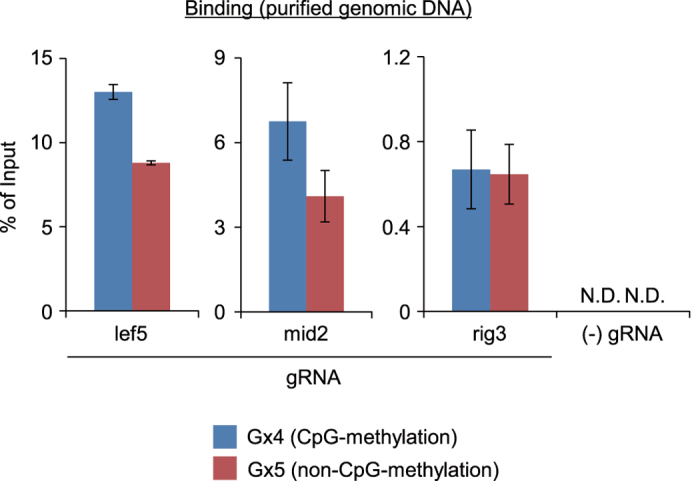
CpG methylation does not directly suppress binding of CRISPR to purified DNA. Genomic DNA was purified from HCT116 cells and used for *in vitro* enChIP; DNA yields of enChIP are shown. Error bars represent s.e.m. of three *in vitro* enChIP experiments. N.D.: not detected.

Next, we investigated whether the allelic locus-binding preference of sgRNA_rig3 was not directly affected by CpG methylation. To this end, we employed *in vitro* enChIP technology using recombinant CRISPR ribonucleoproteins (RNPs)^32^. In *in vitro* enChIP, target genomic regions can be isolated without loss of the molecular interactions in cells that do not express the CRISPR complex^32^. This technology can be applied to sequence-specific isolation of target DNA from purified genomic DNA (Supplementary Fig. S4)^32^. In this study, we performed *in vitro* enChIP with CRISPR RNA (crRNA) : trans-activating crRNA (tracrRNA) duplex instead of sgRNA. As shown in Fig. 3, when *in vitro* enChIP was performed with gRNA_lef5 (crRNA_lef5 : tracrRNA) or gRNA_mid2 (crRNA_mid2 : tracrRNA) using purified genomic DNA (which retains its characteristic *in vivo* methylation patterns) from HCT116 cells, CpG methylation did not suppress binding of CRISPR to the target site (Fig. 3). This result was consistent with the observed binding of CRISPR to the target site *in vivo* (Fig. 2c). Moreover, *in vitro* enChIP with gRNA_rig3 (crRNA_rig3 : tracrRNA) resulted in comparable DNA yields of the target site between the CpG-methylated Gx4 and non-CpG-methylated Gx5 alleles, suggesting that CpG methylation had no suppressive effect on binding of CRISPR to purified genomic DNA *in vitro*. This result is in sharp contrast to the allelic locus-binding preference of the CRISPR complex containing sgRNA_rig3 *in vivo* (Fig. 2c). Thus, the allelic preference of locus binding and genome editing by each sgRNA reflects the locus accessibility of the target sites *in vivo*, which may be influenced by nucleosome positioning, chromatin structure, occupancy by DNA-binding molecules, and other factors.

### Analysis of allele-specific locus binding of the CRISPR complex in another locus *in vivo*

**Figure 4:**
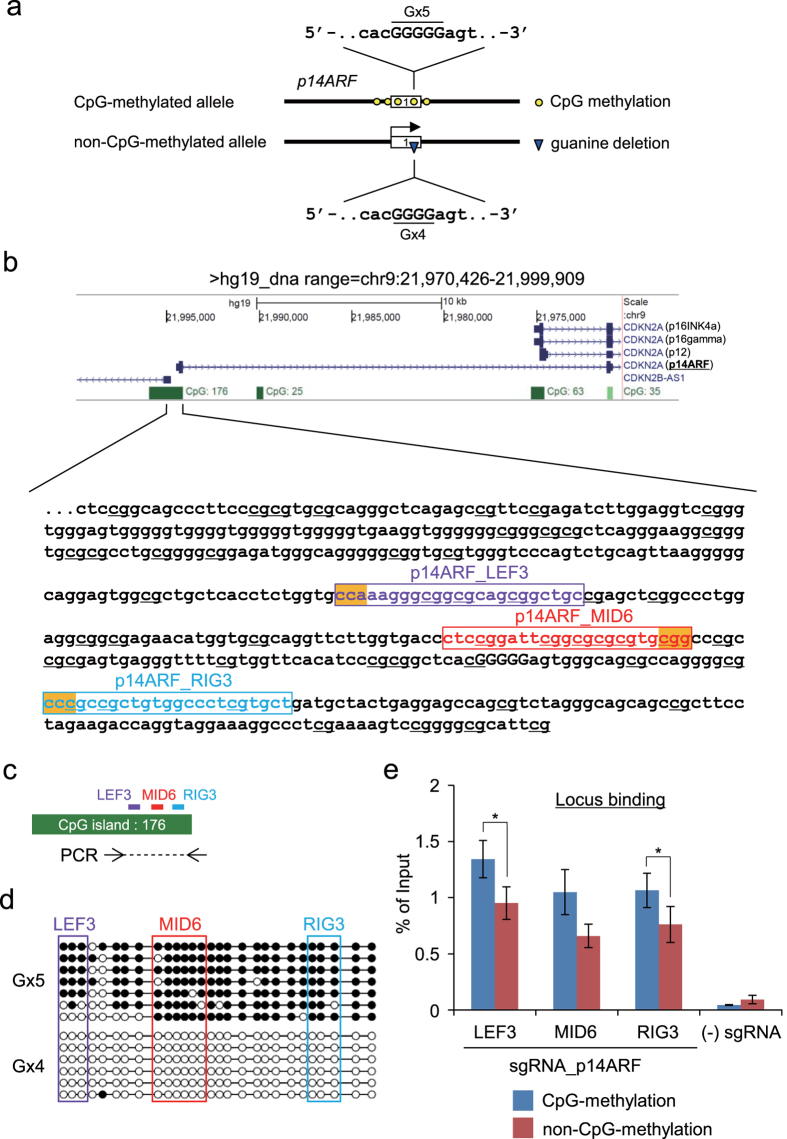
Evaluation of *p14ARF* locus binding by CRISPR *in vivo*. (**a**) Structure of the human *p14ARF* gene in HCT116. One allele of the human *p14ARF* gene is not transcribed in HCT116 because the CpG island (including the promoter region and first exon) is CpG-methylated. In the other allele, a frameshift mutation caused by deletion of a single guanine in the coding region of the first exon prevents production of the functional protein. (**b**) The CpG island of the *p14ARF* gene in HCT116. (Upper) Schematic diagram of the CpG island around the first exon of *p14ARF*. The CpG islands are shown in green. (Lower) A partial DNA sequence of the CpG island (CpG: 176) in the methylated allele. A guanine (G) is deleted from the G stretch (shown in uppercase) of the non-methylated allele. The upper image and DNA sequence were generated using the UCSC Genome Browser (https://genome.ucsc.edu/). CpG sites are underlined. The target sites for sgRNA_p14ARF_LEF3, sgRNA_p14ARF_MID6, and sgRNA_p14ARF_RIG3 are shown in purple, red, and light blue, respectively. PAMs are shown in yellow. (**c**) Primer positions for bisulfite sequencing. (**d**) Bisulfite sequencing of genomic DNA extracted from HCT116. Target sites for sgRNAs are constitutively CpG-methylated in an allele-specific manner. (**e**) DNA yields of conventional enChIP. enChIP targeting *p14ARF* was performed similarly to Supplementary Fig. S3a. Error bars represents s.e.m. of three enChIP experiments (*t-test P-value < 0.05).

It is interesting to know how easily we can find an sgRNA suitable for allele-specific locus binding of CRISPR in an imprinted locus. To this end, we also examined allele-specific locus binding of CRISPR to the *p14ARF* gene, which is also allele-specifically CpG-methylated and silenced at the level of transcription^33^ (Fig. 4a–d). We designed three sgRNAs targeting the CpG island in the gene; two sgRNAs target DNA sequences containing three CpG sites similarly to sgRNA_rig3 (Fig. 4b–d). Although two sgRNAs (p14ARF_LEF3 and p14ARF_RIG3, Fig. 4b) showed statistically significant difference in allelic preference (Fig. 4e and Supplementary Fig. S5), the difference (~1.4-fold higher DNA yields for CpG-methylated allele, Fig. 4e) was much smaller than that with sgRNA_rig3 for the *p16INK4a* locus (~5-fold difference, Fig. 2c). Thus, although allele-specific locus binding of CRISPR is possible with a suitable sgRNA such as sgRNA_rig3 for *p16INK4a*, it might not be easy to find sgRNAs with large allelic preference. More effort and trial-and-error approaches would be required to find a logical designing strategy for allele-specific applications of CRISPR using different chromatin states.

### Allele-specific genome editing by the CRISPR complex using a single-nucleotide gap form

**Figure 5:**
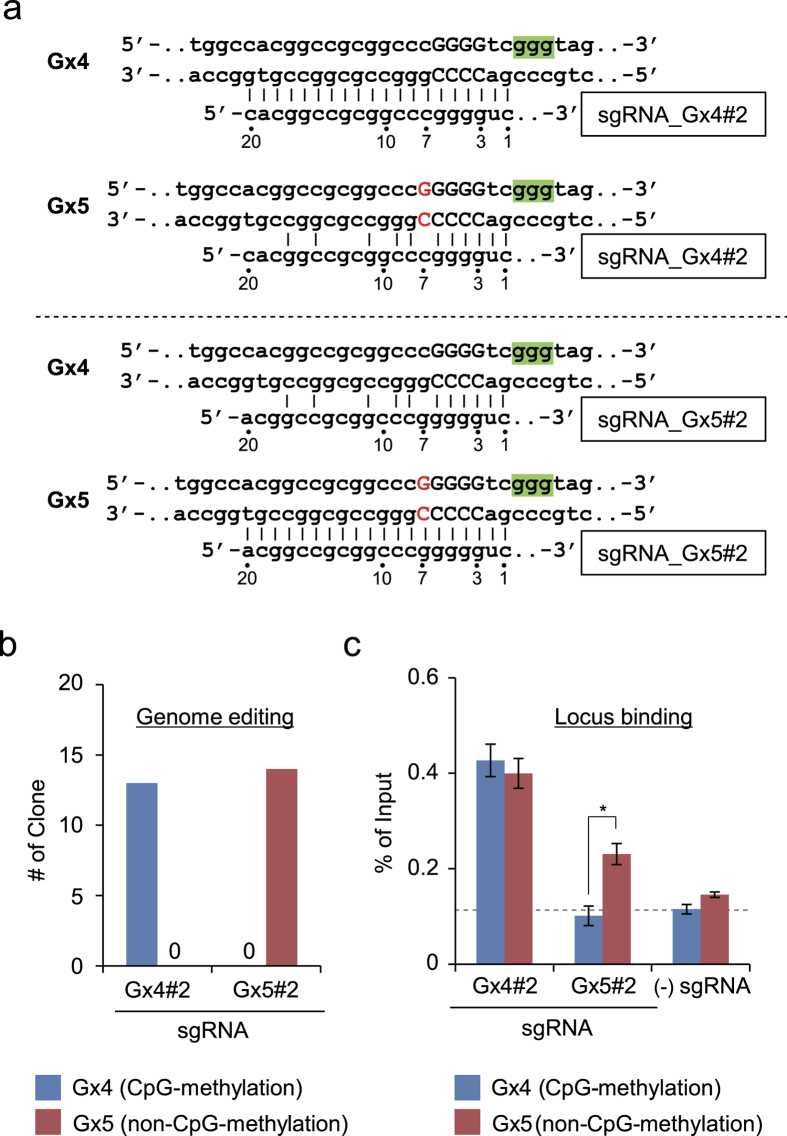
Allele-specific genome editing using an allele-specific single-nucleotide insertion *in vivo*. (**a**) DNA sequences targeted by sgRNAs. PAMs are shown in green. The inserted single guanine in the Gx5 allele is shown in red. (**b**) Evaluation of genome editing. Schemes for genome editing and genotyping PCR are shown in Supplementary Fig. S8. Products of genotyping PCR were cloned, and 13 (sgRNA_Gx4#2) or 14 (sgRNA_Gx5#2) independent clones were subjected to DNA sequencing analysis to identify the targeted alleles. (**c**) Evaluation of locus binding, as determined by DNA yields of conventional *in vivo* enChIP. The error bar represents s.e.m. of three enChIP experiments (*t-test P-value < 0.05).

Allele-specific genome editing by CRISPR can take advantage of SNPs between alleles^14^,^15^. Therefore, using the single-guanine insertion in the *p16INK4a* gene, we next investigated whether a single-nucleotide gap form could also be utilized for allele-specific genome editing by CRISPR *in vivo*. Recent work showed that CRISPR can tolerate sequence mismatches through single-nucleotide skipping in the sgRNA and cleave the mismatched sites^34^,^35^. We confirmed that cleavage of target sites caused by single-nucleotide skipping is observed if the gap occurs at the first or second base 5′ of the PAM (Supplementary Fig. S6a–c). In addition, the CRISPR complex tolerated sequence mismatches at the locus-binding step (Supplementary Fig. S6d). In light of the results of the aforementioned reports^34^,^35^, skipping between the third and seventh bases 5′ of the PAM is unlikely to occur or to be less effective in tolerating sequence mismatches even if it occurs. Therefore, we designed two sgRNAs specifically targeting the Gx4 (sgRNA_Gx4#2) or Gx5 (sgRNA_Gx5#2) position to avoid undesirable single-nucleotide skipping at the first or second base 5′ of the PAM and allow it between the third and seventh bases (Fig. 5a and Supplementary Fig. S7). As shown in Fig. 5b and Supplementary Fig. S8, Gx4 and Gx5 allele-specific genome editing occurred successfully using sgRNA_Gx4#2 and sgRNA_Gx5#2, respectively. These results clearly showed that an allele-specific single-nucleotide gap form can be used for allele-specific genome editing by CRISPR if the gRNA includes the gap form between the third and seventh bases 5′ of the PAM.

**Figure 6:**
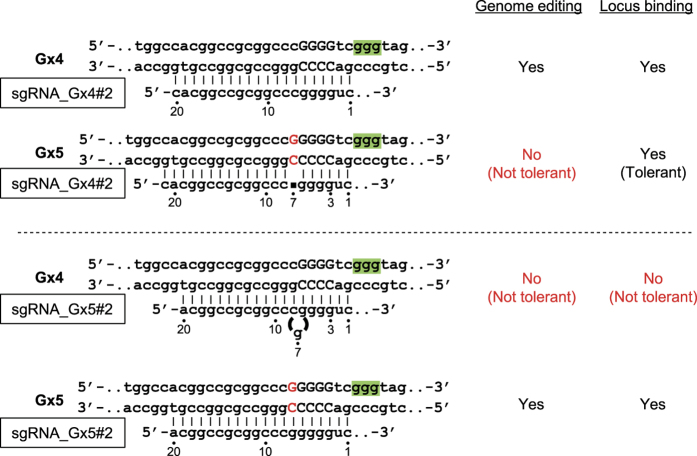
Summary of genome editing and locus binding using sgRNA_Gx4#2 or sgRNA_Gx5#2. Single-nucleotide skipping [“sgRNA jump (DNA bulge)” for sgRNA_Gx4#2 and “sgRNA bulge” for sgRNA_Gx5#2] can occur between the third and seventh positions 5′ of the PAM for each sgRNA. As a representative, single-nucleotide skipping at the seventh nucleotide 5′ of the PAM is shown for each sgRNA. PAMs are shown in green. The single-guanine insertion in the Gx5 allele is shown in red.

We also investigated whether a single-nucleotide gap form could also be utilized for allele-specific locus binding by CRISPR *in vivo*. As shown in Fig. 5c, dCas9 in complex with sgRNA_Gx5#2 bound to the target site in a Gx5 allele-specific manner, consistent with the results of genome editing (Fig. 5b). This result showed that an allele-specific single-nucleotide gap form can also be used for allele-specific locus binding by CRISPR. By contrast, sgRNA_Gx4#2 induced recruitment of dCas9 to both alleles equally (Fig. 5c), suggesting that allele-specific genome editing occurs through different molecular mechanisms for sgRNA_Gx4#2 and sgRNA_Gx5#2: sgRNA_Gx4#2 distinguishes the single-guanine insertion after the locus-binding step, whereas sgRNA_Gx5#2 distinguishes it at the binding step (Fig. 6). Therefore, the reported single-nucleotide skipping rule^34^,^35^ might apply to allele-specific genome editing, but not necessarily to allele-specific locus binding.

## Discussion

In this study, focusing on allele-specific applications of CRISPR, we first examined the effects of chromatin states at target sites on CRISPR-mediated locus binding as well as on genome editing *in vivo* (Figs 2 and 3). Although the allele-specific locus binding and genome editing were achieved only by sgRNA_rig3, our results revealed the following issues concerning the targeting of CpG-methylated sites for these applications: (1) CpG methylation does not necessarily affect locus binding or genome editing by CRISPR *in vivo*, although we are currently unable to eliminate the possibility that the position and number (>5) of methylated cytosines (mCs) in a target site might change this viewpoint. (2) Allelic preference may simply be determined by the accessibility of target sites in each allele, rather than by direct effects of CpG methylation. (3) Conventional *in vivo* enChIP technology is useful for the evaluation of the accessibility of target sites *in vivo*.

It has been reported that CpG methylation (~4 mCs in a target site) does not affect genome editing by CRISPR using a CpG-methylated pUC19 plasmid *in vitro* and a CpG-methylated endogenous locus *in vivo*^19^. Because our conclusions are consistent with this observation, the lack of a direct effect of CpG methylation on CRISPR*-*mediated genome editing may be considered a general phenomenon. In addition, we also showed that CpG methylation (~5 mCs in a target site) does not directly suppress locus binding by CRISPR (Fig. 3). To our knowledge, this study is the first report to investigate the effects of CpG methylation on locus binding of CRISPR *in vivo* and *in vitro* while focusing on the allele-specific applications of CRISPR.

CpG methylation of target sequences interferes with DNA recognition by TAL proteins^36^,^37^. However, because the TAL DNA-binding module that recognizes thymine also recognizes mC^36^,^37^, this module could be applied to the targeting of CpG-methylated sequences^38^. In this study, in addition to the *in vivo* observations, we showed that CpG methylation of purified genomic DNA did not directly affect target binding by CRISPR (Fig. 3). Therefore, it is not necessary to consider the direct effects of CpG methylation of target sites when CRISPR is used for applications such as genome editing and locus binding. However, in the context of allele-specific applications, the aforementioned properties of TAL proteins (i.e., the ability to distinguish between methylated or un-methylated cytosines) might provide an advantage over the CRISPR system.

We showed a possibility that allele-specific locus binding and genome editing can be achieved by targeting an allele-specifically transcribed locus *in vivo*. However, only sgRNA_rig3 for the *p16INK4a* locus was successful (Figs 1 and 2), and detailed molecular basis for the allele-specific locus binding have not been yet elucidated at this stage. In addition, it remains unclear how we can find sgRNAs suitable for allele-specific targeting using different chromatin states. In this regard, it would be an interesting future study to identify the factors that contribute to the allelic preference of sgRNA_rig3 (see below).

In the Gx4 allele of *p16INK4a*, the exon 1 region contains H3K9m2, a heterochromatin mark^24^. Genomic regions with heterochromatin structures are thought to be closed and inaccessible^12^,^16^,^17^,^18^. Although the *p16INK4a* exon 1 region is heavily methylated, some of the sgRNAs we tested could effectively recruit dCas9 to target sites in the region *in vivo* (Fig. 2c), suggesting that these target sites are locally open even in heterochromatin structures *in vivo*, allowing CRISPR to access these sites. By contrast, sgRNA_rig3 could not recruit dCas9 to the target site in the Gx4 allele (Fig. 2c). Because CpG methylation does not directly affect binding of dCas9, allelic preference might be determined by the local accessibility of the target site *in vivo*, in which nucleosome positioning, chromatin structure, occupancy by DNA-binding molecules, and other factors might play important roles. In this regard, a nucleosome boundary is likely to be present around the target site for sgRNA_mid2 in CpG-methylated *p16INK4a* in the human gastric adenocarcinoma cell line AGS^39^. The CpG-methylated Gx4 allele in HCT116 might be organized in the same way. Thus, it might be possible that nucleosome positioning and/or chromatin structures are involved in the allelic preference of sgRNA_rig3. Alternatively, unidentified DNA-binding molecules might simply occupy the target site of sgRNA_rig3, which would inhibit binding of dCas9. On the other hand, our results suggest that accessibility of target sites *in vivo* in heterochromatin regions can be evaluated by conventional *in vivo* enChIP technology. If target sites prove to be accessible even in heterochromatin regions, they can be targeted for CRISPR-mediated genome editing or other applications, such as locus-specific epigenetic modifications to introduce euchromatinization^40^,^41^.

We succeeded in performing allele-specific CRISPR-mediated genome editing using an allele-specific single-guanine insertion in the *p16INK4a* gene. Thus, in addition to SNPs^14^,^15^, a single-nucleotide gap form between two alleles can be exploited for allele-specific applications of CRISPR *in vivo* (Fig. 5). In contrast to the situation using sgRNA_rig3, we could systematically design sgRNAs suitable for this purpose on the basis of the previous reports^34^,^35^. To avoid potential nucleotide skipping, it was necessary to design the sgRNA to include the gap form between the 3rd and 7th bases 5′ from the PAM. Indeed, CRISPR with sgRNA_Gx4 tolerated the single-nucleotide guanine insertion by single-nucleotide skipping, preventing successful allele-specific locus binding and genome editing (Supplementary Fig. S6). Previous reports showed that nucleotide mismatches can be tolerated by CRISPR through single-nucleotide skipping^34^,^35^. However, no previous report has investigated whether single-nucleotide skipping can also occur during locus binding by CRISPR. In this study, we found that the reported rule for single-nucleotide skipping in genome editing^34^,^35^ can be applied to locus binding in some cases (i.e. sgRNA_Gx5#2) (Fig. 5c). We also observed that although single-nucleotide skipping does not occur for nucleotide mismatches between the 3rd and 7th bases 5′ from the PAM during genome editing, skipping occurred even for these mismatches when sgRNA_Gx4#2 but not sgRNA_Gx5#2 was used to specify the locus binding of dCas9 (Fig. 5c). The mode of single-nucleotide skipping obviously differed between these sgRNAs (Fig. 6): specifically, sgRNA_Gx4#2 and sgRNA_Gx5#2 are predicted to engage in “sgRNA jumping (DNA bulge)” and “sgRNA bulge” types of skipping, respectively. Therefore, single-nucleotide skipping by the DNA bulge type might simply tolerate more single-nucleotide mismatches at the locus binding step than at the genome editing step. Alternatively, an alternative PAM “cgg” for sgRNA_Gx4#2 might result in increased mismatch tolerance in locus binding by CRISPR (Supplementary Fig. S9). Indeed, Supplementary Fig. S9 shows that 2-bp mismatches adjacent to PAM could be tolerated, although such off-target sequences would not be effectively tolerated^42^,^43^,^44^,^45^. An additional study will be required to identify the detailed mechanisms.

The CRISPR system can also be used for a wide range of biological applications including artificial transcriptional regulation^2^,^5^,^6^,^9^, epigenetic modification^9^, locus imaging^5^,^6^,^9^, and isolation of specific genomic regions in a locus-specific manner^5^,^9^,^10^. Therefore, defining the rule for single-nucleotide skipping in locus binding would be beneficial for the aforementioned allele-specific applications of CRISPR using a single-nucleotide gap form. Our results might provide information that might be useful for implementing CRISPR in studies of allele-specific genome functions in phenomena such as X-chromosome inactivation, genomic imprinting, and cancer.

## Methods

### Cell lines, plasmids, primers, and donor DNAs

The human colorectal carcinoma cell line HCT116 was obtained from the American Type Culture Collection (ATCC). HCT116 or HCT116-derived cells were maintained in McCoy’s 5A Medium (Thermo Fisher Scientific) with 10% (v/v) fetal bovine serum at 37 °C.

The Cas9 expression plasmid (Addgene #41815) was provided by Dr. George Church via Addgene. The dCas9 expression plasmid 3xFLAG-dCas9/pCMV7.1 (Addgene #47948) was described previously^26^. For construction of the sgRNA_rig3 expression plasmid, a plasmid containing the U6 promoter, target sequence, and gRNA scaffold was synthesized by GeneArt Gene Synthesis (Thermo Fisher Scientific). For construction of other sgRNA expression plasmids, the plasmid gRNA Cloning Vector (*Bbs*I), which contains the U6 promoter, *Bbs*I site, and gRNA scaffold, was synthesized by GeneArt Gene Synthesis, and gRNA sequences were cloned into the *Bbs*I site. Primers and donor ssDNA used in this study are shown in Supplementary Table S1.

### Bisulfite treatment and sequencing

Genomic DNA (400 ng) was subjected to bisulfite treatment with the EZ DNA Methylation-Lightning Kit (Zymo Research). Bisulfite-treated DNA (40 ng) was subjected to PCR with TaKaRa EpiTaq HS (for bisulfite-treated DNA) (Takara Bio). PCR cycles were as follows: 40 cycles of 98 °C for 10 sec, 55 °C for 30 sec, and 72 °C for 1 min. PCR products were cloned into T-vector pMD20 (Takara Bio) and subjected to DNA sequencing analysis with the M13 reverse primer. Methylation status was analyzed by QUMA, a methylation analysis tool (http://quma.cdb.riken.jp/index_j.html).

### Genome editing by CRISPR

HCT116 or HCT116-derived cells (4 × 10^5^ cells) were transfected with 2 μg each of Cas9 expression plasmid, sgRNA expression plasmid, and donor ssDNA using the Lipofectamine 3000 transfection reagent (Thermo Fisher Scientific). To confirm gene targeting, genomic DNA was extracted 2 days after transfection using the *Quick*-DNA Universal Kit (Zymo Research), and then subjected to genotyping PCR with KOD FX (Toyobo). PCR products were cloned into pCR4-TOPO (Thermo Fisher Scientific) and analyzed by DNA sequencing.

### enChIP

HCT116 cells (1.2 × 10^6^ cells) were transfected with 6 μg each of 3xFLAG-dCas9/pCMV7.1 and sgRNA expression plasmid using Lipofectamine 3000. Three days after transfection, the cells were fixed with 1% formaldehyde at 37 °C for 5 min. Chromatin preparation and enChIP were performed as described previously^26^. After isolation of the target genomic regions, DNA was purified using ChIP DNA Clean & Concentrator (Zymo Research).

### Quantitative real-time PCR

Bisulfite-treated DNA was used as the template for real-time PCR with SYBR Premix Ex Taq (Tli RNase H Plus) (Takara Bio) on an Applied Biosystems 7900HT Fast Real-Time PCR System. PCR cycles for *p16INK4a* were as follows: heating at 95 °C for 30 sec; 40 cycles of 95 °C for 5 sec and 62 °C for 1 min. PCR cycles for *p14ARF* were as follows: heating at 95 °C for 30 sec; 35 cycles of 95 °C for 5 sec, 57 °C for 30 sec, and 72 °C for 30 sec.

### *in vitro* enChIP using recombinant CRISPR RNPs

Sonication of purified HCT116 genomic DNA and *in vitro* enChIP using recombinant CRISPR RNPs were performed as described previously^32^. crRNAs and tracrRNA are shown in Supplementary Table S1.

### Statistical analysis

P-values were calculated using the Excel software (Microsoft) using Student’s t-test.

## Additional Information

**How to cite this article**: Fujita, T. *et al*. Allele-specific locus binding and genome editing by CRISPR at the *p16INK4a* locus. *Sci. Rep.* **6**, 30485; doi: 10.1038/srep30485 (2016).

## Supplementary Material

Supplementary Information

## Figures and Tables

**Table 1:** Information on CpG methylation at target sites.

	sgRNA_lef5	sgRNA_mid2	sgRNA_rig3
Target allele	Gx4 and Gx5	Gx4 and Gx5	Gx4 and Gx5
Target position in the CpG island	412–434	542–564	593–615
Total # of CpG	5	2	3
# of CpG in the seed sequence and/or PAM	4	2	2
Allelic preference for genome editing	No	No	Gx5 (non-CpG-methylation)
Allelic preference for binding	No	No	Gx5 (non-CpG-methylation)
