# Supplementary material for: Allele-specific locus binding and genome editing by CRISPR at the p16INK4a locus
Source: Sci Rep. 2016 Jul 28;6:30485. doi: 10.1038/srep30485 (PMC4964623; doi:10.1038/srep30485)
Supplement: Supplementary Information [file srep30485-s1.pdf]

Supplementary Information

Allele-specific locus binding and genome editing by CRISPR at the *p16INK4a* locus

Toshitsugu Fujita, Miyuki Yuno, and Hodaka Fujii

Supplementary Figure Legends

Supplementary Figure S1. Genome editing by CRISPR *in vivo*. (**a**–**c**) (**Left panel**) Schemes for genome editing by CRISPR, and primer positions for genotyping PCR. Wild-type HCT116 (**a** and **c**) or HCT116/del#3 (**b**), a HCT116-derived cell line, was used. Details of HCT116/del#3 are shown in Supplementary Figure S2. (**Right panel**) Genotyping PCR for confirmation of homologous recombination introduced by CRISPR. M: DNA size marker. Products of genotyping PCR were cloned, and independent clones were subjected to DNA sequencing analysis to identify targeted alleles. Results are shown in Figure 2c.

Supplementary Figure S2. Introduction of indels in the *p16INK4a* locus using CRISPR. (**a**) Experimental scheme for introduction of CRISPR-mediated indels in the *p16INK4a* locus in HCT116, and primer positions for genotyping PCR. (**b**) Confirmation of the indel. Genomic DNA was subjected to genotyping PCR. PCR products of clone #3 (arrows) were subjected to DNA sequencing analysis to identify the indel. WT: parental HCT116; M: DNA size marker. (**c**) Deletion mutation identified in the Gx5 allele in clone #3 (referred to as HCT116/del#3 hereafter). (**d**) Experimental scheme and primer positions for RT-PCR and bisulfite-PCR. (**e**) RT-PCR. Total RNA extracted from HCT116 or HCT116/del#3 was used for RT-PCR. The RT-PCR product of HCT116/del#3 (arrow) was subjected to DNA sequencing analysis to identify the transcribed *p16INK4a* allele. (**f**) Confirmation of Gx5 allele-specific transcription of the *p16INK4a* gene in HCT116/del#3. The DNA sequencing trace of the RT-PCR product (arrow in **e**) is shown. (**g**) Bisulfite sequencing of genomic DNA extracted from HCT116/del#3. The CpG methylation states of the target site for sgRNA_mid2 (red square) are the same as those in the parental cell line HCT116.

Supplementary Figure S3. Method for evaluation of locus binding of CRISPR *in vivo*. (**a**) Scheme for enChIP combined with bisulfite treatment followed by methylation-specific PCR (MSP). C: cytosine; U: uracil. (**b**) DNA sequence of the CpG island in the Gx4 allele. The primer positions for MSP are shown in pink (forward) and yellow (reverse). DNA sequences downstream of the CpG island are shown in gray. See also Figure 1b. (**c**) Validation of MSP. Genomic DNA extracted from 293T and HCT116 was subjected to bisulfite treatment followed by MSP. As a methylated DNA control, genomic DNA extracted from 293T was treated with CpG methyltransferase and subjected to bisulfite treatment followed by MSP.

Supplementary Figure S4. Method for evaluation of binding of CRISPR to purified DNA *in vitro*. Scheme for *in vitro* enChIP combined with bisulfite treatment followed by methylation-specific PCR (MSP). C: cytosine; U: uracil.

Supplementary Figure S5. Validation of MSP for evaluation of *p14ARF* locus binding by CRISPR *in vivo*. (**a**) A partial DNA sequence of the CpG island (CpG: 176) in the methylated allele of the *p14ARF* locus. The primer positions for methylation-specific PCR (MSP) are shown in pink (forward) and yellow (reverse). See also Figure 4b. (**b**) Validation of MSP. Genomic DNA extracted from 293T and HCT116 was subjected to bisulfite treatment followed by MSP. As a methylated DNA control, genomic DNA extracted from 293T was treated with CpG methyltransferase and subjected to bisulfite treatment followed by MSP.

Supplementary Figure S6. Genome editing and locus binding using sgRNA_Gx4. (**a**) DNA sequences targeted by sgRNA_Gx4. PAMs are shown in green. The single-guanine insertion in the Gx5 allele is shown in red. (**b**) Potential modes of single-nucleotide skipping

at the 1- or 2-base 5' of the PAM to tolerate nucleotide mismatches. (**c**) Evaluation of genome editing. The scheme for genome editing and genotyping PCR is shown in Supplementary Figure S7. Products of genotyping PCR (Supplementary Fig. S7b) were cloned, and 15 independent clones were subjected to DNA sequencing analysis to identify the targeted alleles. (**d**) DNA yields of conventional enChIP. Error bars represents s.e.m. of three enChIP experiments.

Supplementary Figure S7. Potential modes of single-nucleotide skipping. (**a** and **b**) Potential modes of single-nucleotide skipping at the third or seventh base 5' of the PAM to tolerate nucleotide mismatches: (**a**) sgRNA Gx4#2; (**b**) sgRNA Gx5#2. Single-nucleotide skipping might occur between the third and seventh bases 5' of the PAM. Single-nucleotide skipping at third or seventh base is shown as a representative. PAMs are shown in green. The single-guanine insertion in the Gx5 allele is shown in red.

Supplementary Figure S8. Genome editing by CRISPR *in vivo*. (**a**) Scheme for genome editing by CRISPR, and primer position for genotyping PCR. (**b**–**d**) Genotyping PCR for confirmation of homologous recombination introduced by CRISPR with sgRNA_Gx4 (**b**), sgRNA_Gx4#2 (**C**), or sgRNA_Gx5#2 (**d**). M: DNA size marker. Products of genotyping PCR were cloned, and independent clones were subjected to DNA sequencing analysis to identify targeted alleles. The results are shown in Figure 4b or Supplementary Figure S5c.

Supplementary Figure S9. An alternative PAM "cgg" for sgRNA_Gx4#2 in the *p16INK4a* locus. An alternative PAM is shown in green. The single-guanine insertion in the Gx5 allele is shown in red. In this model, 2-bp mismatches adjacent to the alternative PAM could be tolerated by CRISPR.

Supplementary Materials and Methods

CpG methylation

Genomic DNA (1 µg) of human kidney 293T cells was incubated with CpG methyltransferase (M.*Sss*I) (New England Biolabs) at 37°C for 2 h. DNA was purified by phenol/chloroform extraction and subjected to bisulfite treatment.

Genome editing by CRISPR to establish cell lines

HCT116 cells (4×10^5 cells) were transfected with 2 µg each of Cas9 expression plasmid (Addgene#41815), sgRNA_rig3 expression plasmid, and *p16INK4a* promoter–driven GFP expression plasmid using Lipofectamine 3000. Two days after transfection, GFP-positive cells were sorted as single cells and expanded independently. Indels were confirmed by PCR with KOD FX (Toyobo) followed by DNA sequencing analysis. HCT116 clone #3 (HCT116/del#3) was used in subsequent analyses.

RT-PCR

Total RNA was extracted with Isogen II (Nippongene). RT-PCR was performed as described previously [46], except that AmpliTaq Gold 360 Master Mix (Applied Biosystems) was used.

Supplementary References

46. Fujita, T. & Fujii, H. Species-specific 5’-genomic structure and multiple transcription start sites in the chicken Pax5 gene. *Gene* **477,** 24–31 (2011).

Table S1. Primers, ssDNA donors, and gRNAs used in this study

Number	Name	Sequence (5'→3')	Experiments	Categories
27677	hCDKN2A-Bisul-CpG-free-F	TTTTTAGAGGATTTGAGGGATAGG	Bisulfite-PCR in Figure 1d and Supplementary Figure S2g	Primer
27678	hCDKN2A-Bisul-CpG-free-R	CTACCTAATTCCAATTCCCCTACAAACTTC	Bisulfite-PCR in Figure 1d and Supplementary Figure S2g	Primer
27940	hCDKN2A-Bisul-(M)-F2	TTTAGGTGGGTAGAGGGTTTGTAGC	MSP (Methylation) in Figures 2c, 3, and 5c, and Supplementary Figures S3c and S6d	Primer
27941	hCDKN2A-Bisul-(M)-R2	AAAAAACCTCCCCTTTTTCCGAA	MSP (Methylation) in Figures 2c, 3, and 5c, and Supplementary Figures S3c and S6d	Primer
27942	hCDKN2A-Bisul-(U)-F2	TTTAGGTGGGTAGAGGGTTTGTAGTG	MSP (Unmethylation) in Figures 2c, 3, and 5c, and Supplementary Figures S3c and S6d	Primer
27943	hCDKN2A-Bisul-(U)-R2	CAAAAAACCTCCCCTTTTTCCAA	MSP (Unmethylation) in Figures 2c, 3, and 5c, and Supplementary Figures S3c and S6d	Primer
27889	p16_GFP_KI-genotype-R	GAAGAAGTCGTGCTGCTTCATGT	Genotyping PCR in Supplementary Figures S1 and S8 (all sgRNAs)	Primer
27789	hCDKN2A-Gx4_Gx5-R2	CTGCAAACTTCGTCCTCCAGAGT	Genotyping PCR in Supplementary Figure S1a (sgRNA_lef5)	Primer
27953	p16_GFP_KI-genotype-R3	TCTCGCTGTTCACTACAACCTCA	Genotyping PCR in Supplementary Figure S1b (sgRNA_mid2) and Figure S8 (sgRNA_Gx4, sgRNA_Gx4#2, sgRNA_Gx5#2)	Primer
27888	p16_GFP_KI-genotype-F	CAGTCCGACTCTCCAAAAGGAAT	Genotyping PCR in Supplementary Figure S1c (sgRNA_rig3)	Primer
27175	hCDKN2A-Ex1-F	ACTTCAGGGGTGCCACATTC	RT-PCR in Supplementary Figure S2e (p16INK4a)	Primer
27411	hCDKN2A-Ex1_2-R	CATCATCATGACCTGGATCG	RT-PCR in Supplementary Figure S2e (p16INK4a)	Primer
26460	hGAPDH-Ex8_9-F	TGATGACATCAAGAAGGTGGTGAAG	RT-PCR in Supplementary Figure S2e (GAPDH)	Primer
26461	hGAPDH-Ex8_9-R	TCCTTGGAGGCCATGTGGGCCAT	RT-PCR in Supplementary Figure S2e (GAPDH)	Primer
27952	p16_GFP_HEJ-donor3	GGGCTGGCTGGTCACCAGAGGGgaagaagtcgtgctgcttcatgtccgCGGCTGCGGAGAGGGGGAGAGC	Donor_lef5 in Supplementary Figures S1a	ssDNA donor
27951	p16_GFP_HEJ-donor2	CTGACTGGCTGGCCACGGCCGCGGCgaagaagtcgtgctgcttcatgtcccGGGCGCTGCTGGAGGCGGG	Donor_mid2 in Supplementary Figures S1b and S8	ssDNA donor
27936	p16_GFP_HEJ-donor	TGCGGGCGCTGCTGGAGGCGGGGGCGCTGCgaattcacatgaagcagcacgacttcttcCGGAGGCCGATCCAGGTGGGTAGAGGGTCT	Donor_rig3 in Supplementary Figures S1c	ssDNA donor
-	hp16#236_258-crRNA-sp2	UGGGGCGGACCGCGUGCGCUGUUUUAGAGCUAUGCUGUUU	crRNA for gRNA_lef5 in Figure 3	gRNA
-	hp16#Gx5-crRNA-sp2	CACCUCCUCUACCCGACCCCGUUUUAGAGCUAUGCUGUUU	crRNA for gRNA_mid2 in Figure 3	gRNA
-	hp16#1-crRNA-sp2	ACCGUAACUAUUCGGUGCGUGUUUUAGAGCUAUGCUGUUU	crRNA for gRNA_rig3 in Figure 3	gRNA
-	tracrRNA_15-89	AACAGCAUAGCAAGUUAAAAUAAGGCUAGUCCGUUAUCAACUUGAAAAAGUGGCACCGAGUCGGUGCUUUUUUU	tracrRNA for gRNAs (all) in Figure 3	gRNA
28012	hp14ARF-Bisul-CpG-free-F	TTGTTTATTTTTGGTGTTAAAGGG	Bisulfite-PCR in Figure 4d	Primer
28013	hp14ARF-Bisul-CpG-free-R	TTTCCTACCTAATCTTCTAAAAAAC	Bisulfite-PCR in Figure 4d	Primer
28016	hp14ARF-Bisul-(M)-F	GTTTTCGTGGTTTATATTTCGC	MSP (Methylation) in Figure 4e and Supplementary Figure S5	Primer
28017	hp14ARF-Bisul-(M)-R	TAAAAAACGACTACTACCCTAAACG	MSP (Methylation) in Figure 4e and Supplementary Figure S5	Primer
28018	hp14ARF-Bisul-(U)-F	GGGTTTTTGTGGTTTATATTTTGTG	MSP (Unmethylation) in Figure 4e and Supplementary Figure S5	Primer
28019	hp14ARF-Bisul-(U)-R	AAAAACAACTACTACCCTAAACACT	MSP (Unmethylation) in Figure 4e and Supplementary Figure S5	Primer

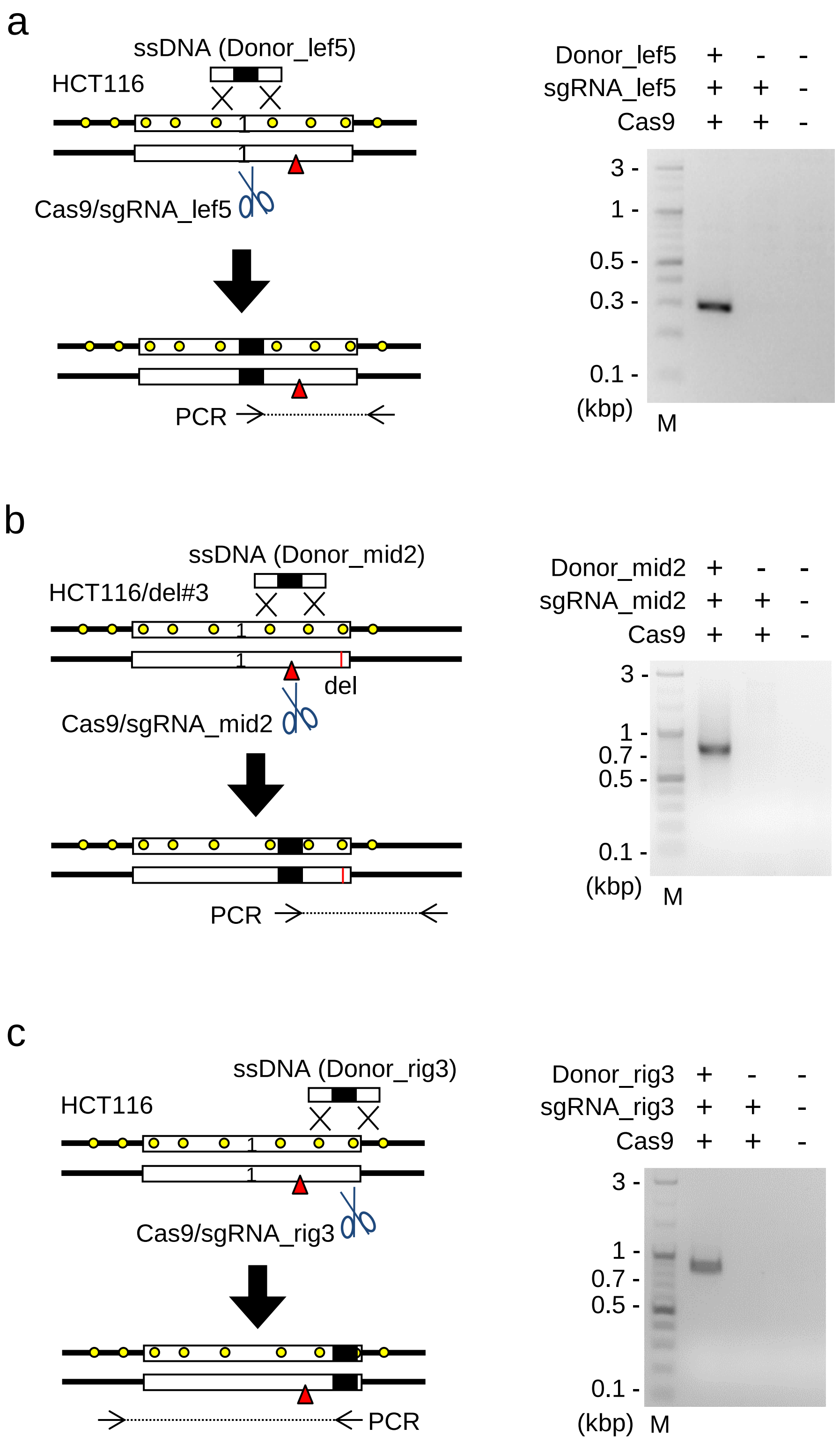

Supplementary Figure S1

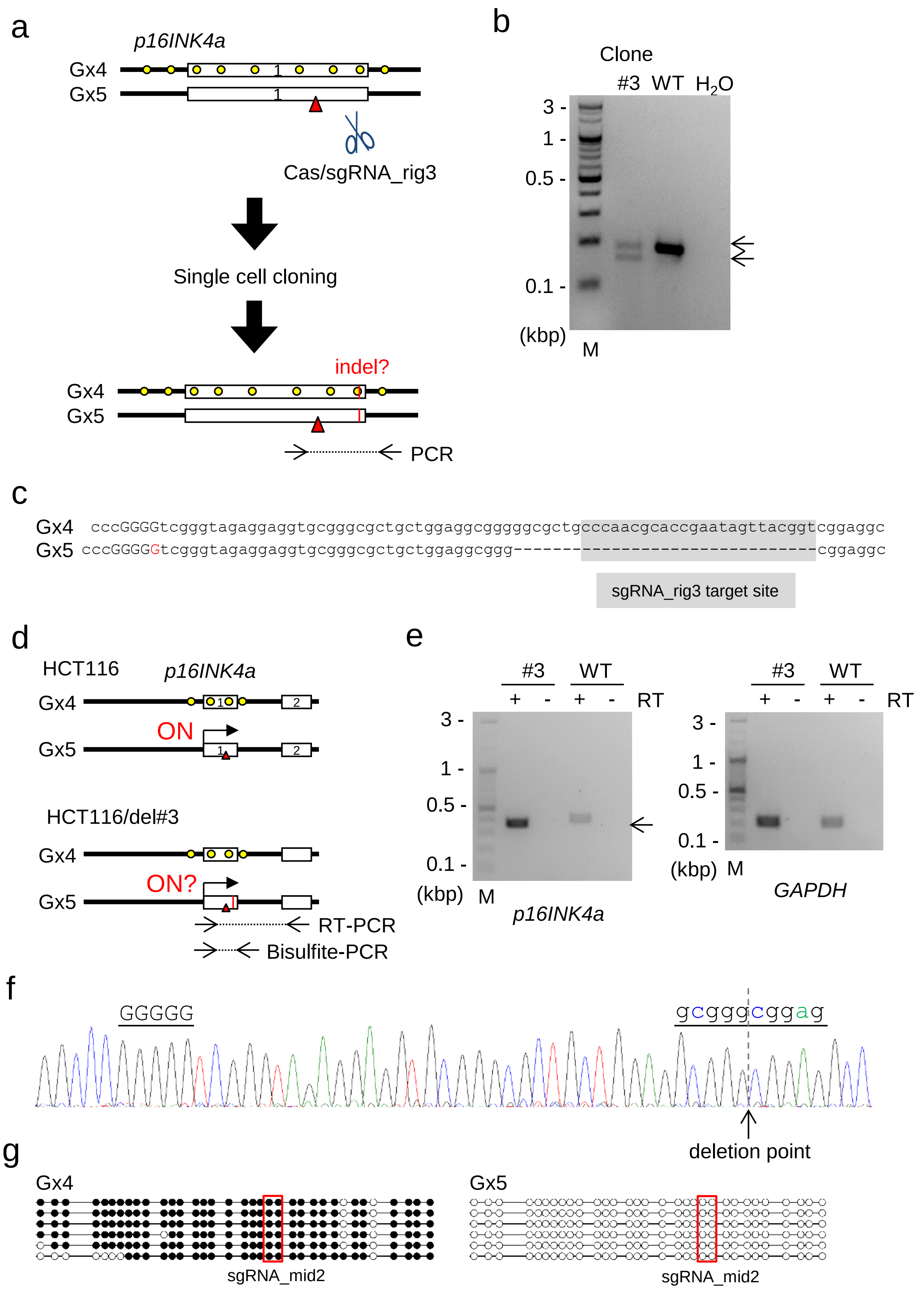

Supplementary Figure S2

a

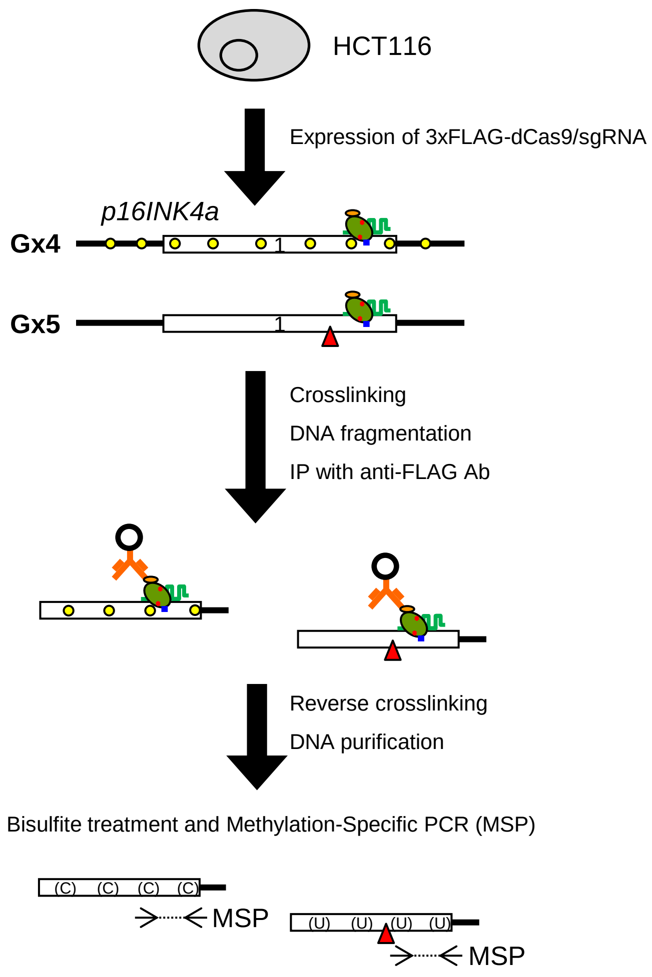

c

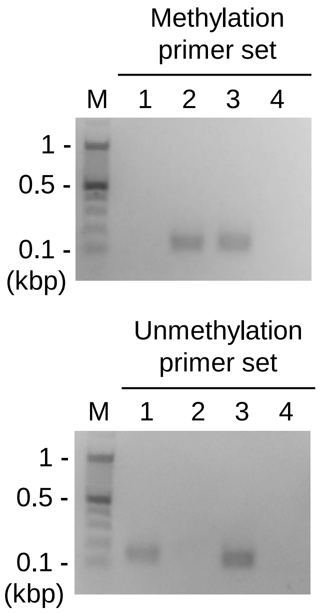

M. DNA size marker
1. 293T gDNA
2. 293T gDNA with CpG methylation
3. HCT116 gDNA
4. H_2O

b

cgcggaggaaggaaacggggcgggggcggatttctttttaacagagtgaacgcactcaaacacgcctt
tgctggcaggcgggggagcgcggctgggagcagggaggccggagggcggtgtgggggcaggtgggga
ggagcccagtcctccttccttgccaacgctggctctggcgagggctgcttccggctggtgccccggg
ggagacccaacctggggcgacttcaggggtgccacattcgctaagtgctcggagttaatagcacctcc
tccgagcactcgctcacggcgtccccttgcctggaaagataccgcggtccctccagaggatttgaggg
acagggtcggagggggctcttccgccagcaccggaggaagaaagaggaggggctggctggtcaccaga
gggtggggcggaccgcgtgcgctcggcggctgcggagagggggagagcaggcagcgggcggcggggag
cagcatggagccggcggcggggagcagcatggagccttcggctgactggctggccacggccgcggccc
GGGGtcgggtagaggaggtgcgggcgctgctggaggcgggggcgctgcccaacgcaccgaatagttac
ggtcggaggccgatccaggtgggtagagggtctgcagcgggagcaggggatggcgggcgactctggag
gacgaagtttgcaggggaattggaatcaggtagcgcttcgattctccggaaaaaggggaggcttcctg

Supplementary Figure S3

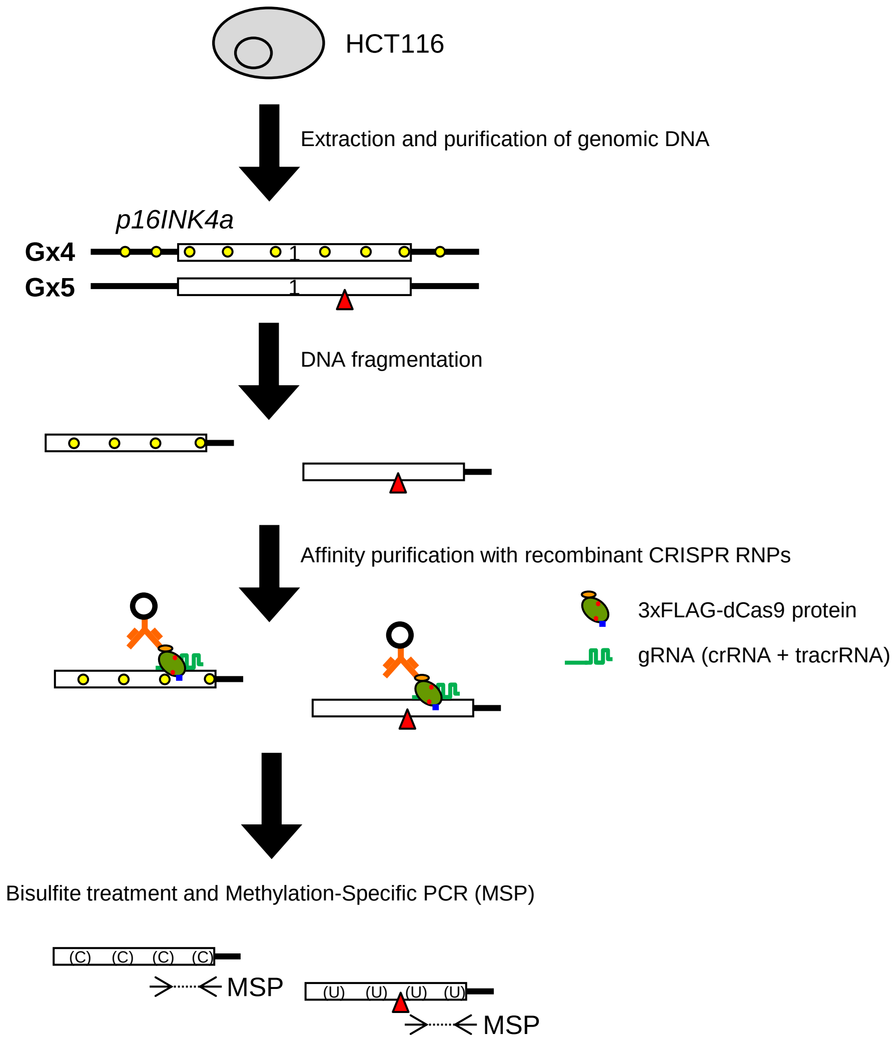

Supplementary Figure S4

a

...ctccggcagcccttcccgcgtgcgcagggctcagagccgttccgagatcttggaggtccggg
tgggagtgggggtggggtgggggtgggggtgaaggtgggggcgggcgcgctcagggaaggcggg
tgcgcgcctgcggggcggagatgggcagggggcggtgcgtgggtcccagtctgcagttaagggg
caggagtggcgctgctcacctctggtgccaaagggcggcgcagcggctgccgagctcggccctgg
aggcggcgagaacatggtgcgcaggttcttggtgaccctccggattcggcgcgcgtgcggcccgc
cgcgagtgagggttttcgtggttcacatcccgcgggctcacGGGGagtgggcagcgccaggggcg
cccgccgctgtggccctcgtgctgatgctactgaggagccagcgtctagggcagcagccgcttcc
tagaagaccaggtaggaaaggccctcgaaaagtccggggcgcattcg

b

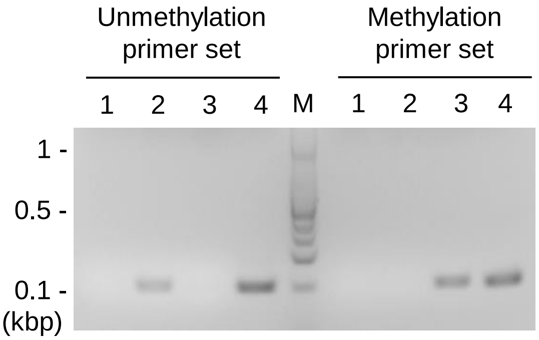

M. DNA size marker
1. H_2O
2. 293T gDNA
3. 293T gDNA with CpG methylation
4. HCT116 gDNA

Supplementary Figure S5

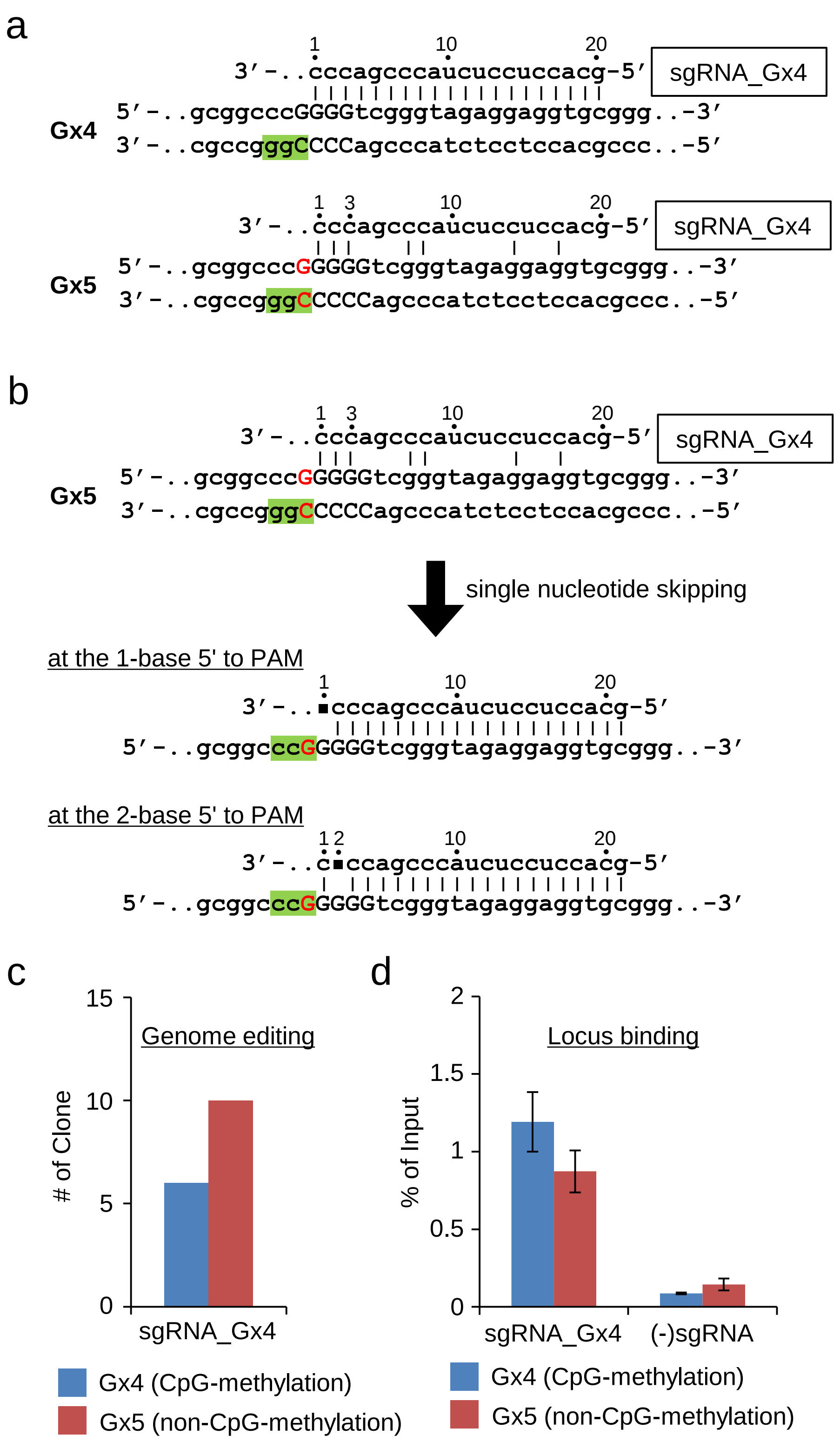

Supplementary Figure S6

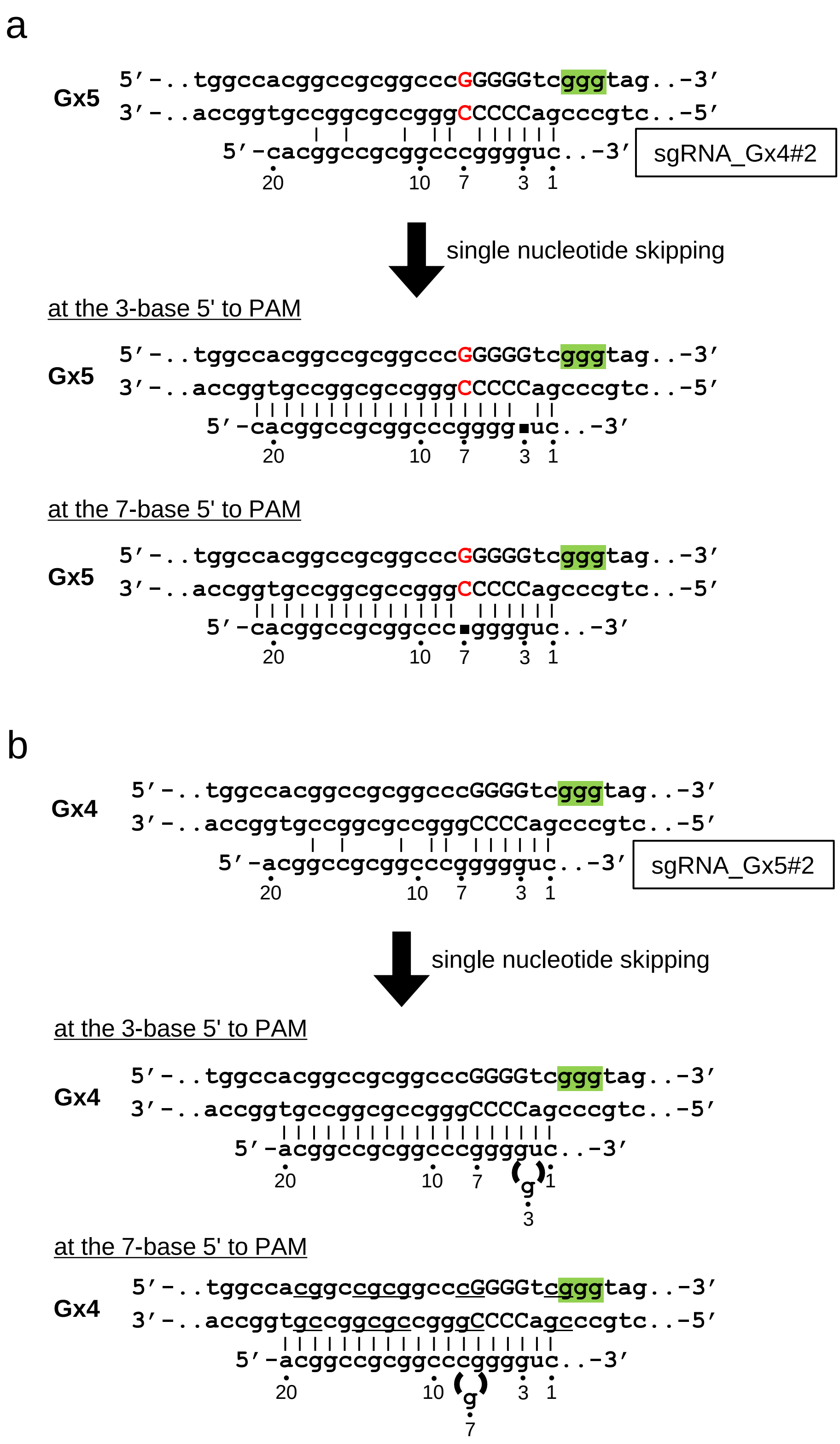

Supplementary Figure S7

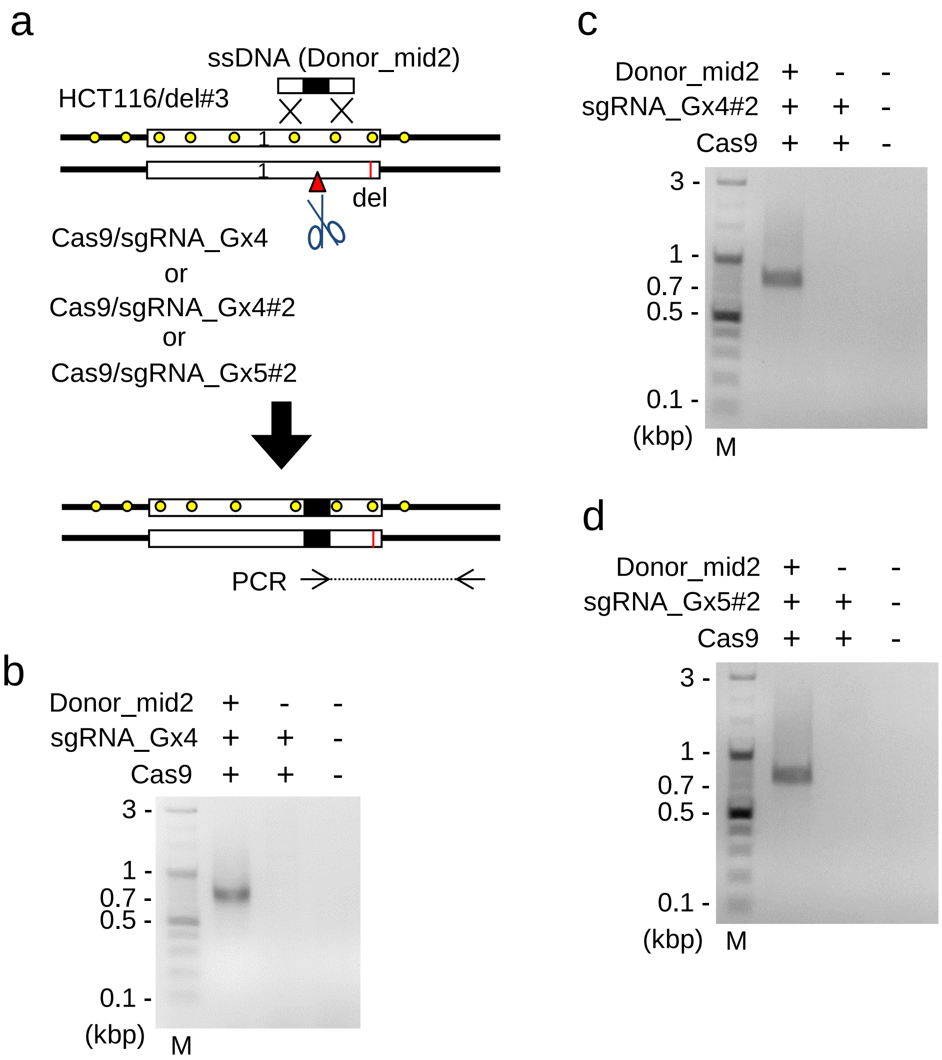

Supplementary Figure S8

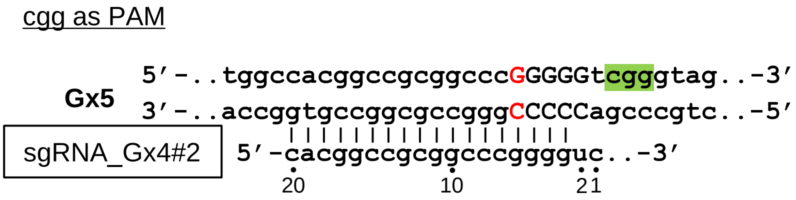

Supplementary Figure S9
